# Novel loci for hyperglycemia identified by QTL mapping of longitudinal phenotypes and congenic analysis

**DOI:** 10.1038/s41598-023-28189-9

**Published:** 2023-01-24

**Authors:** Naru Babaya, Michiko Itoi-Babaya, Hironori Ueda, Misato Kobayashi, Shinsuke Noso, Yoshihisa Hiromine, Akira Ishikawa, Tomomi Fujisawa, Hiroshi Ikegami

**Affiliations:** 1 Department of Endocrinology, Metabolism and Diabetes, Kindai University Faculty of Medicine, 377-2 Ohno-Higashi, Osaka-Sayama, Osaka, 589-8511 Japan; 2 Health Care Center, Rinku General Medical Center, Osaka, Japan; 3 Department of Molecular Endocrinology, Osaka University Graduate School of Medicine, Osaka, Japan; 4 Health Care Center, KSC Branch, Kwansei Gakuin University, Hyogo, Japan; 5 Department of Nutritional Sciences, Nagoya University of Arts and Sciences, Aichi, Japan; 6 Laboratory of Animal Genetics and Breeding, Graduate School of Bioagricultural Sciences, Nagoya University, Aichi, Japan; 7 Sakai City Medical Center, Osaka, Japan

**Keywords:** Genetics, Diabetes

## Abstract

We previously reported that four hyperglycemia loci are located on three chromosomes in the Nagoya-Shibata-Yasuda (NSY) mouse model, commonly used to study type 2 diabetes. However, we did not search for hyperglycemia loci across all chromosomes. In this study, we performed quantitative trait loci (QTLs) mapping of longitudinal phenotypes from crosses between NSY (hyperglycemic) and C3H (normoglycemic) mice. We identified four new QTLs for hyperglycemia, namely *Nidd5nsy*, *Nidd6nsy*, *Nidd1c3h*, and *Nidd2c3h*, on Chromosome 1, 4, 10, and 13, respectively. These QTLs were associated with hyperglycemia in young mice and had attenuated effects in older mice. *Nidd5nsy* and *Nidd6nsy* were hyperglycemic with NSY alleles, and *Nidd1c3h* and *Nidd2c3h* were hyperglycemic with C3H alleles. We further bred *Nidd5nsy* congenic mice and demonstrated that *Nidd5nsy* has a strong effect on hyperglycemia when young, accompanied by insulin resistance and visceral fat accumulation. These results showed that the effects of individual QTLs strengthened or weakened with age, and that the sum of the effects of QTLs captured the age-related deterioration of glucose tolerance in individuals. Our results support the importance of longitudinal phenotypes in the genetic analysis of polygenic traits and have implications for the genetic basis and pathogenesis of type 2 diabetes in humans.

## Introduction

Multiple susceptibility genes and environmental factors, as well as their interactions, are involved in the pathogenesis of type 2 diabetes mellitus^1–3^, which makes understanding the genetic basis of type 2 diabetes a formidable challenge. Recent genome-wide association studies (GWAS) of type 2 diabetes in humans have mapped a number of susceptibility loci, but the effects of these variants only account for a part of disease heritability^4,5^. Furthermore, it is often difficult to identify disease-causing genes or variants in humans due to heterogeneity in genetic backgrounds and environments. To overcome these limitations in human subjects, it is important to use animal models that are physiologically and genetically close to humans, thus minimizing the confounding effects of genetic heterogeneity and uncontrollable environments. Nagoya-Shibata-Yasuda (NSY) mice were established as an inbred animal model of type 2 diabetes by selecting mice with impaired glucose tolerance from a closed colony of Jcl:ICR mice^6^. The NSY mouse phenotype resembles that of human type 2 diabetes, in that the onset is age-dependent, the mouse is moderately obese, and shows impaired insulin responsiveness to glucose. Insulin resistance contributes to the onset, and environmental factors influence the incidence of diabetes^6,7^. In contrast to NSY mice, C3H mice exhibit normal glucose tolerance and have been used in many studies, including this one. The phenotype of C3H mice is well analyzed and the strain is non-obese, insulin sensitive, and has excellent insulin secretion^8,9^.

In an earlier study, we mapped four major quantitative trait loci (QTLs) for impaired glucose tolerance and related phenotypes in NSY mice: *Nidd1nsy*, *Nidd2nsy*, *Nidd3nsy*, and *Nidd4nsy* to chromosomes (Chr) 11, 14, 6, and 11, respectively^10^. Among them, Chr11 and Chr14 were responsible for the development of hyperglycemia at 48 weeks of age, explaining approximately half of the genetic variance^10^. Subsequent studies using consomic mice possessing diabetogenic Chr11 or Chr14 from NSY mice on the genetic background of control C3H mice clearly demonstrated that the genes responsible for hyperglycemia were located on Chr11 and Chr14. However, double consomic mice possessing both Chr11 and Chr14 from NSY mice did not exhibit the diabetic phenotypes of NSY mice^11,12^, indicating that additional genes are responsible for the remaining genetic variance.

In our initial study, we identified the loci *Nidd1nsy-Nidd4nsy* using a two-step approach^10^. Namely, a subpopulation of F2 mice (93 out of 307 mice) exhibiting extreme phenotypes in glucose tolerance were subjected to initial genome screening with a full panel of markers, and three significant chromosomes (Chr11, Chr14, and Chr6) were further analyzed in the whole population of F2 mice. However, this approach prevented the analysis of the remaining chromosomes in detail. Therefore, the aim of this study was to identify new QTLs, besides those on Chr11, Chr14, and Chr6, by analyzing the QTL mapping data of the whole F2 population (307 mice) with sufficient genetic markers and longitudinally obtained phenotypes. Furthermore, we aimed to produce a congenic strain possessing the target chromosomal region containing the newly identified loci and analyze the glucose tolerance and other metabolic traits of these mice longitudinally.

## Results

### Novel hyperglycemia loci detected by QTL analysis

**Figure 1:**
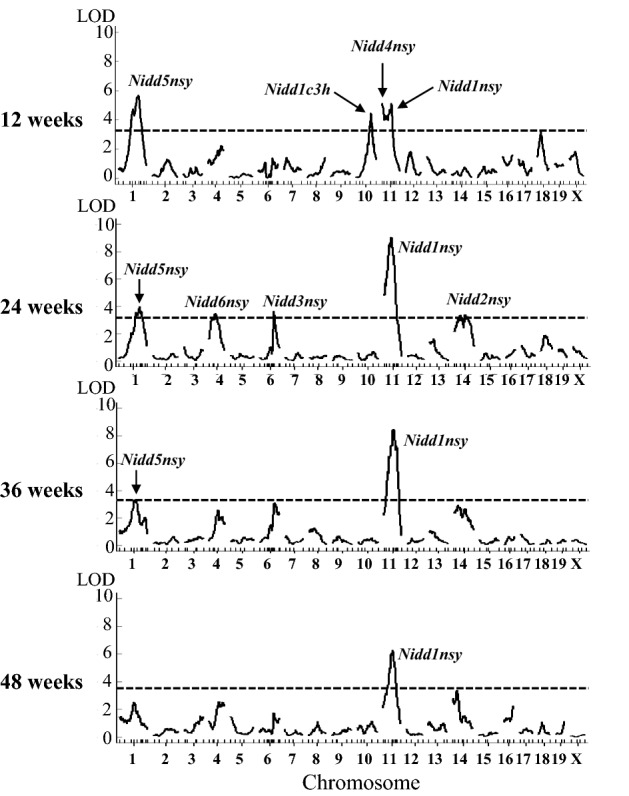
Logarithm of odds (LOD) score curves for the area under the glucose curve (gAUC) after glucose challenge at 12, 24, 36, and 48 weeks of age. LODs were calculated using the R/qtl package. The x-axis represents chromosome positions from the centromere; the y-axis shows LOD scores. The dashed line in each graph represents the threshold LOD value obtained from multiple imputation approach at α = 0.05 by applying 1000 permutations for each trait. These values were 3.21, 3.33, 3.24, and 3.28 for 12, 24, 36, and 48 weeks of age, respectively.

QTL mapping of the F2 progeny (n = 307) using the R/qtl package identified four novel loci for diabetes on Chr1, Chr4, Chr10, and Chr13, in addition to reconfirming the previously identified *Nidd1nsy-Nidd4nsy* (Tables 1, 2, Fig. 1, and Supplementary Table S1). In contrast to the previously identified *Nidd1nsy* on Chr11, which was linked to hyperglycemia at all ages, the effects of the newly identified loci were age-dependent, with significant linkages observed at younger ages, but not older ages (Table 1, Fig. 1, and Supplementary Table S1). The NSY-derived alleles for loci on Chr1 and Chr4 were positively linked to hyperglycemia. Meanwhile, for loci on Chr10 and Chr13, the C3H-derived alleles were positively linked to hyperglycemia. Therefore, loci on Chr1 and Chr4 were designated *Nidd5nsy* and *Nidd6nsy* for non-insulin-dependent diabetes mellitus loci 5 and 6 in NSY mice, respectively. However, the loci on Chr10 and Chr13 were designated *Nidd1c3h* and *Nidd2c3h* for non-insulin-dependent diabetes mellitus locus 1 and 2 in C3H mice, because they caused hyperglycemia in C3H mice and the NSY allele had a protective effect against hyperglycemia.

**Table 1:** Maximum LOD scores for diabetes-related phenotypes in the detected QTLs.

	Locus							
	*Nidd1nsy * (Chr11)	*Nidd2nsy * (Chr14)	*Nidd3nsy * (Chr6)	*Nidd4nsy * (Chr11)	*Nidd5nsy * (Chr1)	*Nidd6nsy * (Chr4)	*Nidd1c3h * (Chr10)	*Nidd2c3h * (Chr13)
Age (weeks)								
Phenotypes								
12								
Glucose: 0 min	–	–	–	–	6.14	–	3.89	4.41
Glucose: 30 min	–	–	–	–	3.79	–	5.17	–
Glucose: 60 min	4.63	–	–	4.65	4.72	–	3.25	–
Glucose: 90 min	5.38	–	–	4.71	5.27	–	3.43	–
Glucose: 120 min	4.40	–	–	5.46	4.97	–	–	–
gAUC	5.11	–	–	5.13	5.68	–	4.46	–
Body weight	–	–	–	–	–	–	–	–
24								
Glucose: 0 min	–	–	–	–	–	–	–	–
Glucose: 30 min	7.25	–	–	4.48	4.84	–	–	–
Glucose: 60 min	7.53	–	3.98	#	3.95	3.46	–	–
Glucose: 90 min	8.75	3.58	3.45	#	3.41	–	–	–
Glucose: 120 min	7.99	4.74	3.95	#	–	–	–	–
gAUC	8.94	3.51	3.67	#	4.19	3.40	–	–
Body weight	–	–	–	–	–	–	–	–
36								
Glucose: 0 min	4.13	–	–	–	–	–	–	–
Glucose: 30 min	8.87	–	–	–	4.27	–	–	–
Glucose: 60 min	7.90	–	3.61	–	–	–	–	–
Glucose: 90 min	7.41	–	3.40	–	–	–	–	–
Glucose: 120 min	7.09	–	–	–	–	–	–	–
gAUC	8.45	–	–	–	3.39	–	–	–
Body weight	–	–	–	–	–	–	–	–
48								
Glucose: 0 min	3.91	–	–	–	–	–	–	–
Glucose: 30 min	5.08	–	–	–	–	–	–	–
Glucose: 60 min	5.91	–	–	–	–	–	–	–
Glucose: 90 min	6.58	3.55	–	–	–	–	–	–
Glucose: 120 min	6.31	3.59	–	–	–	–	–	–
gAUC	6.45	–	–	–	–	–	–	–
Body weight	–	–	–	–	–	–	–	–
52								–
Epididymal fat pads	–	3.43	6.93*^1^	–	–	–	–	–

Maximum LOD scores calculated by R/qtl are shown. Threshold LOD values obtained using the multiple imputation approach at α = 0.05 are between 3.18 and 3.41, and at α = 0.01 are between 3.76 and 4.22.

*AUC* area under the curve.

– Data not significant; # omitted because there was no obvious peak and heavily influenced by *Nidd1nsy*. *^1^This locus overlaps with *Fn1n*^17^.

**Table 2:** Summary of hyperglycemia QTLs in NSY with C3H mice.

	Locus															
	*Nidd1nsy * (Chr11)				*Nidd2nsy * (Chr14)		*Nidd3nsy * (Chr6)	*Nidd4nsy * (Chr11)			*Nidd5nsy * (Chr1)		*Nidd6nsy * (Chr4)	*Nidd1c3h * (Chr10)		*Nidd2c3h * (Chr13)
Nearest marker	*D11Mi242*				*D14Mit59*		*D6Mit135*	*D11Mit76*			*D1Mit14*		*D4Mit219*	*D10Mit230*		*D13Mit134*
Position of the QTL*^1^ (Physical position *^2^)	26.4–49.4 cM (39–51 Mbp)				13.5–71.5 cM (21–89 Mbp)		61.7–75.7 cM (124–138 Mbp)	9.4–50.4 cM (12–52 Mbp)			51.7–90.7 cM (75–160 Mbp)		14.0–62.0 cM (31–122 Mbp)	52.0–78.0 cM (56–88 Mbp)		4.5–36.5 cM (11–39 Mbp)
Hyperglycemia phenotype with highest LOD score	gAUC				Glucose: 120 min		Glucose: 60 min	Glucose: 120 min			Glucose: 0 min		Glucose: 60 min	Glucose: 30 min		Glucose: 0 min
The age (weeks)	24				24		24	12			12		24	12		12
Effect size of nearest marker (mean ± SD)																
NSY/NSY allele	2004.1 ± 504.7**^††^				14.4 ± 6.4**^††^		18.7 ± 6.0**	10.1 ± 3.8**^††^			3.7 ± 0.8**^††^		18.8 ± 5.3**	12.3 ± 2.8**^††^		3.1 ± 0.7**^†^
NSY/C3H allele	1717.3 ± 516.8**				11.8 ± 5.7		18.3 ± 5.1**	8.4 ± 3.2			3.4 ± 0.8*		18.2 ± 6.0**	14.3 ± 3.6		3.4 ± 0.7
C3H/C3H allele	1505.3 ± 523.2				10.2 ± 4.3		15.4 ± 5.8	7.7 ± 2.5			3.1 ± 0.7		15.7 ± 5.2	15.4 ± 3.3		3.6 ± 0.9
Unit	mmol/L × min				mmol/L		mmol/L	mmol/L			mmol/L		mmol/L	mmol/L		mmol/L
Allele number in the nearest marker																
NSY/NSY allele	84				82		79	77			75		92	56		72
NSY/C3H allele	153				151		153	136			140		143	139		111
C3H/C3H allele	70				72		72	74			84		72	66		86
Mode of inheritance *^3^	Additive				Additive		Dominant	Additive			Additive		Additive	Additive		Additive
Variance explained (%)	12.8				6.8		5.7	8.3			8.1		5.2	7.8		6.5
Number of genes *^4^	110				671		210	243			516		688	428		282

*^1^95% confidence interval (CI), corresponding to a 1.8-LOD support interval.

*^2^Physical positions were estimated from marker positions.

*^3^Mode of inheritance of the NSY allele was determined using the degree of dominance, which is the ratio of the dominance effect to the additive effect (see “Materials and methods” section).

*^4^Number of genes in the 95% CI was estimated using the Mouse Genome Database (http://www.informatics.jax.org).

Values were compared using one-way ANOVA with post hoc test (Bonferroni): *P < 0.05, **P < 0.01 vs CC; ^†^*P* < 0.05, ^††^*P* < 0.01 vs NC.

### Characteristics of the hyperglycemia locus on Chr1: *Nidd5nsy*

The *Nidd5nsy* locus on Chr1, which was mapped to the region near *D1Mit14* (95% confidence interval [CI]: 75–160 Mbp)*,* was strongly linked to fasting blood glucose levels at 12 weeks of age (maximum logarithm of odds [LOD] score [MLS]: 6.14, P < 0.01) (Table 1). This locus was also strongly linked to blood glucose levels at 30, 60, 90, and 120 min after the intraperitoneal glucose tolerance test (ipGTT) (MLS: 3.79, 4.72, 5.27, 4.97, and P: < 0.05, < 0.01, < 0.01, < 0.01, respectively) (Table 1). Further, the area under the glucose curve (gAUC) at 12 weeks of age was strongly linked to this locus (MLS: 5.68, P < 0.01) (Table 1 and Fig. 1). *Nidd5nsy* explained 8.1% of the phenotypic variance of fasting glucose at 12 weeks of age (Table 2). F2 mice possessing NSY-derived alleles at *D1Mit14* had significantly higher gAUC values than C3H-derived alleles (P < 0.01) (Supplementary Table S1). At 24 weeks of age, *Nidd5nsy* was linked to glucose levels at 30, 60, and 90 min post-challenge and gAUC (MLS: 4.84, 3.95, 3.41, 4.19, and P: < 0.01, < 0.05, < 0.05, < 0.05, respectively) (Table 1). The effect of *Nidd5nsy* diminished with age and, at 48 weeks of age, *Nidd5nsy* was not linked to glucose levels. This locus was most strongly linked to hyperglycemia at 12 weeks of age, followed by 24 weeks of age, indicating that *Nidd5nsy* especially affects glucose tolerance at a young age. No markers tested on Chr1 were linked to body weight at any age nor epididymal fat pad weight collected at 52 weeks of age.

### Confirmation of newly identified *Nidd5nsy* on Chr1 by congenic strain analysis

To directly demonstrate the existence of *Nidd5nsy* on Chr1 and localize and characterize the gene, we constructed a congenic strain possessing the NSY-derived *Nidd5nsy* region of Chr1 on the control C3H background. The constructed congenic mice, C3H.NSY-*Nidd5nsy*, carried NSY-derived alleles for Chr1 from *D1Mit305* to *D1Mit269*, including the region of interest (Supplementary Fig. S1).

**Figure 2:**
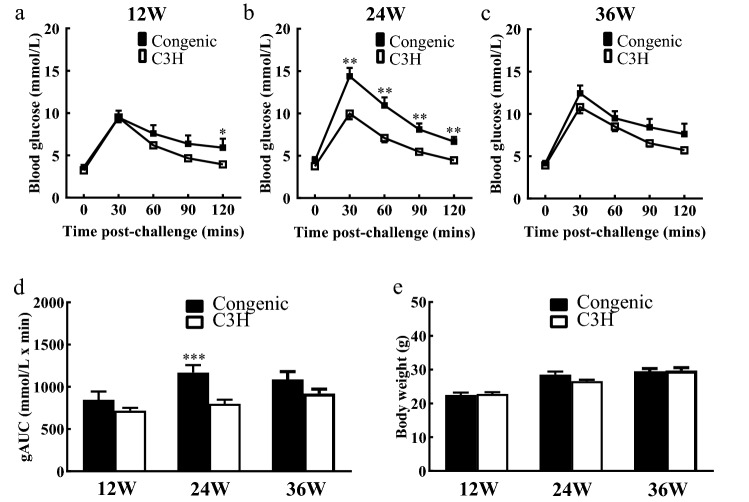
Longitudinal phenotypes of congenic C3H.NSY-*Nidd5nsy* and C3H mice. (**a–c**) Blood glucose levels after glucose challenge at (**a**) 12, (**b**) 24, and (**c**) 36 weeks of age. Closed squares, C3H.NSY-*Nidd5nsy* mice; open squares, C3H mice. (**d**) Area under the glucose curve (gAUC) after glucose challenge. (**e**) Body weight. The number of mice at 12, 24, and 36 weeks of age were 21, 20, and 23 in C3H.NSY-*Nidd5nsy* mice and 14, 22, and 10 in C3H mice, respectively. Data represent the mean ± SEM. *P < 0.05, **P < 0.01, ***P < 0.001.

The results from ipGTT and body weights of congenic C3H.NSY-*Nidd5nsy* and C3H mice are shown in Fig. 2. At 24 weeks of age, glucose levels were significantly higher in C3H.NSY-*Nidd5nsy* mice than in C3H mice at all time points post glucose challenge (P < 0.01) (Fig. 2b). gAUC values were also significantly higher in C3H.NSY-*Nidd5nsy* mice than in C3H mice (P < 0.001) (Fig. 2d). At 12 weeks of age, glucose levels at 120 min post glucose challenge were significantly higher in C3H.NSY-*Nidd5nsy* mice than in C3H mice (P < 0.05); however, no significant difference was observed at 36 weeks of age, indicating that *Nidd5nsy* had an age-dependent effect on hyperglycemia. There were no significant differences in body weight between C3H.NSY-*Nidd5nsy* and C3H mice at any age (Fig. 2e).

At 24 weeks of age, the most significant differences were observed in the glucose tolerance test, so the diabetes-related phenotype of C3H.NSY-*Nidd5nsy* mice was compared to that of C3H mice at 26–28 weeks of age (Table 3 and Supplementary Fig. S2). Insulin secretion (at 26 weeks of age), assessed by the insulinogenic index after glucose challenge, was similar for C3H.NSY-*Nidd5nsy* and C3H mice. HOMA-IR, an index of insulin resistance, was significantly higher for C3H.NSY-*Nidd5nsy* mice than for C3H mice (P < 0.05). Additionally, in a separate group from the ipGTT considered for dissection (at 28 weeks of age), C3H.NSY-*Nidd5nsy* mice exhibited significantly higher body weights (P < 0.01), BMI (P < 0.05), and visceral fat weights (P < 0.05) than C3H mice, suggesting that increases in body fat and insulin resistance were the main causes of hyperglycemia induced by *Nidd5nsy*. The development of hyperglycemia in the face of insulin resistance with normal insulin secretion suggests the failure of insulin secretion to compensate for insulin resistance.

**Table 3:** Phenotypes in congenic C3H.NSY-*Nidd5nsy* and C3H mice.

		C3H.NSY-*Nidd5nsy*	C3H^c^
Diabetes index (26 weeks)		n = 12	n = 21
Insulin secretion^a^		8.2 ± 1.6	8.0 ± 0.8
HOMA-IR^b^		155.0 ± 31.2*	84.9 ± 18.2
Anatomical analysis (28 weeks)		n = 8	n = 20
Body weight	(g)	32.8 ± 0.8**	29.5 ± 0.5
Anal-nasal length	(cm)	10.4 ± 0.0*	10.2 ± 0.1
BMI	(g/cm^2^)	0.301 ± 0.006*	0.283 ± 0.005
Total visceral fat	(g)	1.653 ± 0.220*	1.023 ± 0.052
Epididymal fat	(g)	0.838 ± 0.145*	0.509 ± 0.033
Retroperitoneal fat	(g)	0.345 ± 0.048**	0.119 ± 0.009
Mesenteric fat	(g)	0.471 ± 0.040	0.395 ± 0.020
Total visceral fat/body weight	(%)	4.97 ± 0.59*	3.49 ± 0.18

Values represent total number or mean ± SEM. Characteristics were compared using the Mann–Whitney *U* test. *P < 0.05, **P < 0.01.

^a^Assessed by insulinogenic index (incremental insulin area under the curve (AUC) [pmol/L] divided by incremental glucose AUC [mmol/L]) after glucose challenge.

^b^Calculated from basal insulin and glucose concentrations (fasting glucose [mmol/L] × fasting insulin [pmol/L]).

^c^C3H mice data were previously reported^12^.

### Characteristics of hyperglycemia loci on Chr4, Chr10, and Chr13

The *Nidd6nsy* locus on Chr4, which was mapped to the region near *D4Mit219* (95% CI: 31–122 Mbp)*,* was strongly linked to glucose levels at 60 min post glucose challenge (MLS: 3.46, P < 0.05) and gAUC (MLS: 3.40, P < 0.05) at 24 weeks of age (Table 1 and Fig. 1). *Nidd6nsy* explained 5.2% of the phenotypic variance in glucose levels at 60 min post glucose challenge (Table 2). F2 mice with NSY-derived alleles at *D4Mit219* had significantly higher gAUC values than those without these alleles (P < 0.01) (Supplementary Table S1). The *Nidd6nsy* locus was not linked to glucose levels at 12, 36, or 48 weeks of age. No markers tested on Chr4 were linked to body weight at any age nor epididymal fat pad weight collected at 52 weeks of age.

The *Nidd1c3h* locus on Chr10, which was mapped to the region near *D10Mit230* (95% CI: 56–88 Mbp)*,* was strongly linked to blood glucose levels at 0, 30, 60, and 90 min post glucose challenge at 12 weeks of age (MLS: 3.89, 5.17, 3.25, 3.43 and P: < 0.05, < 0.01, < 0.05, < 0.05, respectively) (Table 1). Consequently, gAUC was strongly linked to this locus (MLS: 4.46, P < 0.01) (Table 1 and Fig. 1). F2 mice with NSY-derived alleles at *D10Mit230* had significantly lower, not higher, gAUC values (P < 0.01) (Supplementary Table S1). NSY-derived alleles at *D10Mit230* were not linked to glucose levels at 24, 36, or 48 weeks of age. Additionally, NSY-derived alleles at *D10Mit230* were not linked to body weight at any age nor epididymal fat pad weight collected at 52 weeks of age.

The *Nidd2c3h* locus on Chr13, which was mapped to the region near *D13Mit134* (95% CI: 11–39 Mbp)*,* was only linked to blood glucose levels at fasting glucose at 12 weeks of age (MLS: 4.41, P < 0.01) (Table 1). F2 mice with NSY-derived alleles at *D13Mit134* had a significantly lower glucose value at 0 min (P < 0.01) (Table 2).

### Confirmation of previously identified hyperglycemia loci: *Nidd1nsy–Nidd4nsy*

*Nidd1nsy–Nidd4nsy*, which were identified in our previous study using only 68 markers with the MAPMAKER/QTL program^10^, were reconfirmed in this study using more markers with the R/qtl package with the same data set as before. Although the *Nidd1nsy*–*Nidd4nsy* loci detected in our previous study may not be exactly the same as the present ones, the peaks of the previously detected QTLs are located within the 95% CI of the QTLs detected in the present study. *Nidd1nsy* on Chr11 in the region near *D11Mit242* (95% CI: 39–51 Mbp) was linked to glucose levels at 12, 24, 36, and 48 weeks of age (Table 1 and Fig. 1), similar to the results of our previous study^10^. *Nidd1nsy* was the locus most strongly linked to hyperglycemia at 24, 36, and 48 weeks of age, and the third most strongly linked locus at 12 weeks of age. *Nidd1nsy* was not linked to body weight at any age nor epididymal fat pad weight at 52 weeks of age. *Nidd2nsy* was reconfirmed on Chr14 (Table 1 and Fig. 1). The LOD peak was located near *D14Mit59* (95% CI: 21–89 Mbp) for hyperglycemia and near *D14Mit160* (95% CI: 22–80 Mbp) for epididymal fat pad weight. *Nidd2nsy* was not linked to body weight at any age. *Nidd3nsy* was reconfirmed on Chr6 (Table 1 and Fig. 1). The LOD peak for glucose levels was located near *D6Mit135* (95% CI: 124–138 Mbp), while that for epididymal fad pad weight was located near *D6Mit54* (95% CI: 94–127 Mbp). *Nidd3nsy* was strongly linked to epididymal fat pad weight at 52 weeks of age (MLS: 6.93, P < 0.01) (Table 1). *Nidd3nsy* was not linked to body weight at any age. *Nidd4nsy* was identified in the centromeric region of Chr11 (95% CI: 12–52 Mbp) on the same chromosome but at a different position from *Nidd1nsy*. *Nidd4nsy* was strongly linked to glucose levels at 12 weeks of age (Table 1 and Fig. 1) and was not linked to body weight at any age.

## Discussion

This study identified four novel hyperglycemia QTLs, including *Nidd5nsy* on Chr1, *Nidd6nsy* on Chr4, *Nidd1c3h* on Chr10, and *Nidd2c3h* on Chr13. For *Nidd5nsy* and *Nidd6nsy*, NSY-derived alleles predisposed mice to developing hyperglycemia, while for *Nidd1c3h* and *Nidd2c3h*, the C3H-derived allele predisposed mice to hyperglycemia. Further, these QTLs were linked to hyperglycemia at younger ages, namely, transient hyperglycemia. The previously identified hyperglycemia loci *Nidd1nsy*-*Nidd4nsy* on Chr6, Chr11, and Chr14 were also reconfirmed in this study. The effect of *Nidd5nsy*, the QTL most strongly linked to hyperglycemia among the newly identified loci, was clearly demonstrated in the congenic study. Moreover, the congenic study clarified that *Nidd5nsy* harbors a locus that causes hyperglycemia, insulin resistance, insufficient insulin secretion to compensate for insulin resistance, and visceral obesity.

The effects of four novel QTLs were age-dependent, with significant linkages detected in young ages which diminished at older ages. Generally, diabetes persists or worsens with age unless therapeutic intervention with diet and exercise is provided after onset. Therefore, a single QTL was expected to cause a similar phenomenon, i.e., persistence or worsening with age. However, the longitudinal phenotypes in this study demonstrated that the effects of some QTLs strengthened while others weakened with age, suggesting that summation of the QTL effects resulted in age-related deterioration of glucose tolerance in the individual.

Several questions arose during our study. First, QTL mapping indicated that *Nidd5nsy* on Chr1 was most strongly linked to hyperglycemia at 12 weeks of age, whereas congenic mice exhibited only mild hyperglycemia at 12 weeks of age and significantly increased blood glucose levels at 24 weeks of age. In addition, an increase in body weight, which was not evident in QTL mapping, was detected in the congenic analysis. A possible reason for this discrepancy is that the effect of *Nidd5nsy* may depend on interactions with other genes of the NSY donor line, which were lost during the construction of the congenic line. In other words, *Nidd5nsy* is affected by gene–gene interactions with age-dependent and -independent effects, resulting in overall phenotypes of type 2 diabetes in NSY mice that vary with age. Polygenic disorders, such as type 2 diabetes and obesity, are affected by multiple gene–gene and gene-environment interactions^13–15^. Thus, it is necessary to consider that genes with age-dependent effects may influence these interactions. Second, the increase in visceral fat mass was not evident in QTL mapping, but was detected in the congenic analysis. One of the possible reasons for this discrepancy may be the difference in phenotyping age: 52 weeks in QTL mapping and 28 weeks in the congenic study. Another possibility is that the congenic mice were affected by *Nidd2nsy* on Chr14 and *Nidd3nsy* on Chr6 from C3H-derived alleles, which increased visceral fat^16,17^.

This study mapped a new QTL for hyperglycemia, *Nidd5nsy* on Chr1. The human syntenic region corresponding to the peak LOD score is Chr1q24.2–32.1. This region contains loci for type 2 diabetes that have been reported in several studies listed in the GWAS catalog (https://www.ebi.ac.uk/gwas/home) (Supplementary Table S2)^18–24^. Although there are still many genes in this region, and identifying susceptibility genes or disease-causing variants is a formidable challenge, these syntenic regions in mice and humans provide important information about common pathways and regulatory genes in both species.

In mouse studies, several QTLs for blood glucose were reported in the region identified in this study. A QTL on distal Chr1, *Nob3*, has been identified in genome-wide scans of diabetic NZO with non-diabetic C57BL/6J^25^. On Chr4, some QTLs have been identified in NZO with SJL mice (*Nidd/SJL*)^26,27^, in NZO with several mouse lines^28^, and in NZO with DBA mice (*Nidd/DBA*)^29^. On Chr10, a QTL has been identified in SM/J and A/J recombinant inbred strains (*t2dm1sa*)^30^. On Chr13, no QTL has previously been identified in the region of interest. The data in the present study together with previous reports point to the importance of these chromosomes in conferring susceptibility to hyperglycemia.

*Nidd1c3h and Nidd2c3h* are distinct from other QTLs in that the NSY-derived alleles from diabetes-prone mice are protective, while the C3H-derived alleles from normoglycemic mice confer susceptibility to type 2 diabetes. Since type 2 diabetes is a polygenic disease and each locus was identified in crosses of two strains by the relative strength of the locus on each phenotype, it is possible that some genes may induce opposite effects. For example, previous studies have reported contradictory effects of *Nidd2nsy* and *Nidd3nsy* on visceral fat weight; alleles from disease-prone NSY mice decreased visceral fat pad weight, whereas alleles from disease-resistant C3H mice increased visceral fat pad weight^16,17^. Such loci effects were also previously reported in NOD mice, a well-known animal model of another polygenic disease, type 1 diabetes^31–33^. Loci inducing the opposite effects may arise as revertants, mutations that reverse pathological processes in disease models. The genes and gene products of such loci provide important information for establishing effective methods of disease prevention and intervention.

This study had some limitations. First, the current NSY-derived *Nidd5nsy* congenic region is approximately 85 Mb, which contains more than 500 genes, making it difficult to examine candidate genes at this time. Furthermore, functional studies suggest that hyperglycemia in *Nidd5nsy* congenic mice is due to both insulin resistance and a compensatory defect in insulin secretion in response to insulin resistance, suggesting that two or more genes may be involved rather than only one. To identify susceptibility genes, more regionally restricted congenic analysis is necessary. Second, in this study, we performed QTL analysis across all chromosomes and found several new QTL, each of which accounted for at most 10% of the phenotypic variance, and collectively could not explain the entire phenotypic trait. This suggests that gene–gene and gene–environment interactions may be involved and need further investigation. Third, we used the R/qtl package in the current study^34^, which differed from that used in our previous studies^10,17^, because the previous program could not be used. Despite differences in programs, the results for *Nidd1nsy*-*Nidd4nsy* were almost identical for both studies, indicating the accuracy of the methods used and validating the new QTLs obtained in this study.

The study findings indicate that type 2 diabetes in NSY mice is regulated by the summation of novel QTLs identified in this study and previously reported QTLs (Chr6, Chr11, and Chr14), with each QTL exerting different age-related effects. Furthermore, this study demonstrates the importance of longitudinal analysis of phenotypes during the genetic analysis of polygenic traits.

## Materials and methods

### Animals

NSY mice^6^ were obtained from the Branch Hospital of Nagoya University School of Medicine (Nagoya, Japan). C3H/HeNcrj (C3H) mice were purchased from Charles River Laboratories (Kanagawa, Japan). All mice (including NSY, C3H, F1, F2, and congenic mice) were bred at Osaka University Medical School, housed in an air-conditioned room (22–25 °C) with a 12 h light–dark cycle (6:00–18:00 h), and provided tap water and standard rodent chow diet (Oriental Yeast, Tokyo, Japan) ad libitum. Male mice were used in all experiments. The animal study protocol was approved by the Osaka University Graduate School of Medicine Ethics Committee and complied with the ARRIVE guidelines. All experiments were performed in accordance with relevant guidelines and regulations.

### Construction of F2 mice and phenotypic characterization

As previously described^10,17^, F1 mice were produced by crossing an NSY male and a C3H female, and F1 mice were intercrossed to produce F2 mice. F2 mice (n = 307) underwent an ipGTT (2 g glucose/kg body weight) after overnight fasting, and blood glucose levels were measured at 0, 30, 60, 90, and 120 min at 12, 24, 36, and 48 weeks of age. Blood glucose levels were measured directly by the glucose oxidase method using Glutest E (Sanwa Kagaku Kenkyusho Co. Ltd., Nagoya, Japan). gAUC was calculated according to the trapezoid rule. F2 mice were euthanized by intraperitoneal administration of pentobarbital sodium (Dainippon, Osaka, Japan) at 52 weeks of age. The epididymal fat pads of F2 mice (n = 221) were dissected and weighed. The phenotypes of F2 mice are shown in Supplementary Fig. S3.

### Genotyping of F2 mice and QTL analysis

Genomic DNA, extracted from the liver or tail using the standard phenol–chloroform method, was used as a template for polymerase chain reaction (PCR). Microsatellite marker information was obtained from the Mouse Genome Database (https://www.informatics.jax.org). A total of 107 microsatellite markers were used to cover the whole genome (Supplementary Table S3, Supplementary Fig. S4). The markers were amplified by PCR with non-labeled or labeled primers. Non-labeled PCR products were electrophoresed on 9% polyacrylamide gel and visualized by ethidium bromide staining. Labeled PCR products were electrophoresed on 4% denaturing polyacrylamide gel using an ABI Prism 3100 Genetic Analyzer (Applied Biosystems, Foster City, CA, USA) with GeneScan 350 ROX (Applied Biosystems) as an internal lane size standard. The PCR fragments were measured using GeneScan Analysis software, version 3.5 (Applied Biosystems) and genotyped using Genotyper software, version 3.6 (Applied Biosystems).

Normality tests were performed for all phenotypic traits, and for traits that deviated significantly from normality, Box–Cox transformations were used to bring them as close to normal as possible. QTL analysis was performed using these trait values. QTL analysis by interval mapping was performed using the R/qtl package, using multiple imputation approach to complement missing genotype data^34–36^. An LOD score was calculated at a 1 cM step within each interval across the linkage map constructed. The 95% CI was obtained by a 1.8-LOD drop method. Threshold LOD values were obtained from multiple imputation approach at α = 0.05 and α = 0.01, by applying 1000 permutations for each trait. The mode of inheritance of the QTL was determined by using the degree of dominance, which is the ratio of the dominance effect to the additive effect, as described by Kenney-Hunt et al.^37^. QTL analysis was performed on 307 mice for glucose and body weight and 221 mice for epididymal fat pads.

### Construction of a congenic strain and phenotypic characterization

A novel congenic strain (C3H.NSY-*Nidd5nsy*) was constructed with the genomic region identified in QTL analysis using a marker-assisted speed-congenic method^38^, as previously described^11,12,16,39,40^. Namely, (NSY × C3H) F1 male mice were backcrossed with female C3H mice. Males that were heterozygous for *Nidd5nsy* were selectively mated with female C3H mice and male progeny that were heterozygous for *Nidd5nsy* were used for the next generation. This process was repeated until all markers in the background genome were homozygous for the C3H-derived genome (N6 or N7). Subsequently, mice heterozygous for *Nidd5nsy* were intercrossed to obtain mice homozygous for *Nidd5nsy*. This line was maintained by brother-sister mating.

Genomic DNA, extracted from the tail using the standard phenol–chloroform method, was used as a template for PCR. A total of 84 marker loci spanning the whole genome were analyzed (Supplementary Table S4). Eleven markers on Chr1 were used to confirm recombination. The marker typing methods were the same as those used for QTL mapping, as described above. The constructed congenic strain, C3H.NSY-*Nidd5nsy*, carried the NSY allele for Chr1 from *D1Mit305* to *D1Mit269* and the C3H allele from *D1Mit215* to centromere and from *D1Mit353* to telomere (Supplementary Fig. S1).

The glucose tolerance and body weights of C3H.NSY-*Nidd5nsy* mice were measured at 12, 24, and 36 weeks of age. The glucose tolerance was assessed by an ipGTT as mentioned above. Insulin secretion in response to glucose was assessed by ipGTT (2 g glucose/kg body weight) in overnight-fasted mice at 26 weeks of age. Blood glucose and plasma insulin levels were measured at 0, 15, and 30 min. Plasma insulin levels were measured using an enzyme-linked immunosorbent assay (ELISA) kit (Morinaga, Yokohama, Japan). Incremental glucose (ΣΔ glucose) and insulin (ΣΔ insulin) levels were calculated from measurements at 0, 15, and 30 min. The insulinogenic index was calculated as ΣΔ insulin divided by ΣΔ glucose, as previously described^11,12,40^. The homeostasis model assessment of insulin resistance (HOMA-IR) was calculated from basal insulin and glucose concentrations.

Congenic mice for dissection were euthanized at 28 weeks of age by intraperitoneal administration of pentobarbital sodium (Dainippon). These mice were in a different group from those used for the glucose tolerance test described above. Body weight, anal-nasal length, body mass index (BMI), and weights of epididymal fat pads, mesenteric fat pads, and retroperitoneal fat were measured.

### Statistical analysis

All data are expressed as mean ± SEM or mean ± SD, as noted in the respective locations. Statistical analysis was performed using the Mann–Whitney U test or one-way ANOVA with post hoc test (Bonferroni). Statistical significance was set at P < 0.05. Statistical analysis was performed using GraphPad Prism, version 9.5.0, statistical software (San Diego, California).

## Supplementary Information

Supplementary Information.

## Data Availability

The datasets used and/or analyzed during the current study available from the corresponding author on reasonable request.
