# Supplementary material for: Novel loci for hyperglycemia identified by QTL mapping of longitudinal phenotypes and congenic analysis
Source: Sci Rep. 2023 Jan 24;13:1315. doi: 10.1038/s41598-023-28189-9 (PMC9873599; doi:10.1038/s41598-023-28189-9)
Supplement: Supplementary file 1 — Supplementary Information. [file 41598_2023_28189_MOESM1_ESM.pdf]

Novel loci for hyperglycemia identified by QTL mapping of longitudinal phenotypes and congenic analysis

Naru Babaya[1], Michiko Itoi-Babaya[2], Hironori Ueda[3,4], Misato Kobayashi[5],

Shinsuke Noso[1], Yoshihisa Hiromine[1], Akira Ishikawa[6], Tomomi Fujisawa[7],

and Hiroshi Ikegami[1]

[1]Department of Endocrinology, Metabolism and Diabetes, Kindai University Faculty of Medicine, Osaka, Japan; [2]Health Care Center, Rinku General Medical Center, Osaka, Japan; [3]Department of Molecular Endocrinology, Osaka University Graduate School of Medicine, Osaka, Japan; [4]Health Care Center, KSC branch, Kwansei Gakuin University, Hyogo, Japan; [5]Department of Nutritional Sciences, Nagoya University of Arts and Sciences, Aichi, Japan; [6]Laboratory of Animal Genetics and Breeding, Graduate School of Bioagricultural Sciences, Nagoya University, Aichi, Japan; [7]Sakai City Medical Center, Osaka, Japan

Supplementary Table S1. Area under the glucose curve after the intraperitoneal glucose tolerance test in detected QTL loci

	Chromosome	marker	alleles		
			NSY/NSY	NSY/C3H	C3H/C3H
12 weeks					
Nidd1nsy	Chr11	*D11Mit242*	1478.1±405.1**†	1353.2±364.2**	1194.6±348.9
Nidd4nsy	Chr11	*D11Mit76*	1507.0±417.1**††	1335.2±371.6	1235.7±328.3
Nidd5nsy	Chr1	*D1Mit14*	1508.7±412.9**††	1358.1±366.3*	1221.7±329.2
Nidd1c3h	Chr10	*D10Mit230*	1162.7±318.3**††	1400.0±407.4	1454.3±372.1
24 weeks					
Nidd1nsy	Chr11	*D11Mit242*	2004.1±504.7**††	1717.3±516.8**	1505.3±523.2
Nidd2nsy	Chr14	*D14Mit59*	1929.9±561.1**††	1719.2±543.4	1603.9±476.9
Nidd3nsy	Chr6	*D6Mit135*	1832.5±568.7**	1787.5±499.9**	1536.8±537.5
Nidd5nsy	Chr1	*D1Mit14*	1959.7±544.7**††	1710.9±539.2	1620.3±504.0
Nidd6nsy	Chr4	*D4Mit219*	1839.8±529.6**	1786.2±568.4**	1552.4±463.6
36 weeks					
Nidd1nsy	Chr11	*D11Mit242*	2139.5±536.0**††	1913.6±550.8**	1632.7±515.6
Nidd5nsy	Chr1	*D1Mit14*	2022.6±536.7*	1914.2±584.6	1821.0±560.0
48 weeks					
Nidd1nsy	Chr11	*D11Mit242*	1921.2±537.1**	1871.3±553.2**	1515.4±498.4

Values are represented as mean ± SD. Units are expressed as mmol/L × min. For *Nidd2c3h*, no QTL for area under the glucose curve was detected at any age; it is therefore not included in this table. Values were compared using one-way ANOVA with post hoc test (Bonferroni): $^{*}P < 0.05$, $^{**}P < 0.01$ vs CC; $^{\dagger}P < 0.05$, $^{\dagger\dagger}P < 0.01$ vs NC.

Supplementary Table S2. Type 2 diabetes loci identified by genome-wide association studies in humans corresponding to mouse *Nidd5nsy* region in this study

Variant and risk allele	P-value	Mapped genes	Human chromosome 1 location	References*
rs111973643-T	2×10^{-6}	*FCGR2A, RNU6-481P*	161,419,933	(1)
rs7412314-?	4×10^{-6}	*FMO4*	171,318,113	(2)
rs4916253-G	3×10^{-9}	*PIGC, DNM3*	172,391,892	(3)
rs7546252-G	2×10^{-10}	*DNM3, PIGC*	172,399,170	(3)
rs633715-C	6×10^{-11}	*LINC01741, SEC16B*	177,883,445	(4)
rs532504-A	7×10^{-12}	*SEC16B*	177,909,798	(5)
rs539515-C	2×10^{-10}	*LINC01741, SEC16B*	177,919,890	(6)
rs539515-C	5×10^{-15}	*LINC01741, SEC16B*	177,919,890	(3)
rs545608-G	7×10^{-10}	*SEC16B, CRYZL2P-SEC16B*	177,929,986	(3)
rs2816177-G	7×10^{-9}	*COX5BP8, SOAT1*	179,279,817	(3)
rs4129858-G	4×10^{-9}	*LAMC1*	183,035,199	(3)
rs1327123-C	7×10^{-9}	*TSEN15, COLGALT2*	184,045,459	(5)
rs1327123-G	7×10^{-10}	*TSEN15, COLGALT2*	184,045,459	(3)
rs12128213-G	2×10^{-9}	*LINC00862, CCNQP1*	200,228,410	(3)
rs567185-C	2×10^{-14}	*NAV1, IPO9-AS1*	201,794,371	(3)
rs41304257-G	8×10^{-12}	*IPO9*	201,880,798	(3)
rs201297151-CAAAAAAAA	3×10^{-8}	*PIK3C2B, MDM4*	204,505,454	(5)
rs12041243-G	3×10^{-10}	*MDM4*	204,521,342	(3)
rs6679717-?	3×10^{-13}	*RNA5SP74, LRRN2*	204,591,549	(7)
rs11240351-G	1×10^{-10}	*CNTN2*	205,075,211	(3)
rs12048743-G	4×10^{-9}	*DSTYK*	205,145,745	(6)
rs12048743-G	2×10^{-12}	*DSTYK*	205,145,745	(3)
rs7538321-T	1×10^{-9}	*SLC41A1, PM20D1*	205,820,327	(3)

*(1) Mansour Aly, D. *et al. Nat. Genet.* **53**: 1534-1542 (2021); (2) Hamet, P. *et al. J. Hypertens*. **35 Suppl 1**: S24-S32 (2017); (3) Vujkovic, M *et al. Nat. Genet.* **52**: 680-691 (2020); (4) Sakaue, S. *et al. Nat. Genet.* **53**(10): 1415-1424 (2021); (5) Spracklen, C.N. *et al. Nature* **582**: 240-245 (2020); (6) Mahajan, A. *et al. Nat. Genet.* **50**:1505-1513 (2018); (7) Ray, D. *et al. PLOS Genet.* **16**: e1009218 (2020).

Supplementary Table S3. Polymorphic markers used in the QTL study

Chromosome	Marker	(cM)	Chromosome	Marker	(cM)	Chromosome	Marker	(cM)	Chromosome	Marker	(cM)
**Chr 1**	*D1Mit64*	(3.67)	**Chr 6**	*D6Mit1*	(6.68)	**Chr 10**	*D10Mit28*	(3.04)	**Chr 15**	*D15Mit13*	(1.84)
	D1Mit173	(24.04)		*D6Mit223*	(26.14)		*D10Mit3*	(17.64)		*D15Mit113*	(27.74)
	D1Mit19	(50.15)		*D6Mit17*	(36.20)		*D10Mit194*	(31.26)		*D15Mit144*	(37.42)
	D1Mit305	(61.58)		*D6Mit209*	(37.44)		*D10Mit230*	(64.56)		*D15Mit42*	(81.04)
	D1Mit218	(73.17)		*D6Mit69*	(39.72)		*D10Mit164*	(86.52)			
	D1Mit14	(88.53)		*D6Mit178*	(45.98)				**Chr 16**	*D16Mit88*	(9.23)
	D1Mit269	(92.84)		*D6Mit64*	(52.09)	**Chr 11**	*D11Mit76*	(9.41)		*D16Mit4*	(26.18)
	D1Mit353	(96.86)		*D6Mit54*	(57.47)		*D11Mit229*	(17.46)		*D16Mit30*	(33.98)
	D1Mit461	(109.17)		*D6Mit366*	(57.80)		*D11Mit236*	(30.30)		*D16Mit158*	(49.94)
	D1Mit155	(119.68)		*D6Mit300*	(60.81)		*D11Mit242*	(43.61)			
				D6Mit52	(64.06)		*D11Mit320*	(48.70)	**Chr 17**	*D17Mit113*	(8.14)
**Chr 2**	*D2Mit176*	(3.79)		*D6Mit135*	(65.15)		*D11Mit195*	(57.97)		*D17Mit36*	(25.28)
	D2Mit296	(25.08)		*D6Mit14*	(84.30)		*D11Mit70*	(62.87)		*D17Mit206*	(45.26)
	D2Mit37	(57.28)		*D6Mit15*	(87.73)		*D11Mit145*	(66.80)		*D17Mit129*	(62.66)
	D2Mit304	(76.89)					*D11Mit168*	(81.95)			
	D2Mit22	(107.32)	**Chr 7**	*D7Mit81*	(29.34)				**Chr 18**	*D18Mit68*	(11.96)
				D7Mit62	(47.23)	**Chr 12**	*D12Mit37*	(2.59)		*D18Mit35*	(26.33)
**Chr 3**	*D3Mit117*	(1.96)		*D7Mit238*	(60.00)		*D12Mit2*	(21.22)		*D18Mit7*	(59.32)
	D3Mit268	(11.05)		*D7Mit259*	(101.95)		*D12Mit51*	(47.56)			
	D3Mit98	(49.39)					*D12Mit141*	(67.48)	**Chr 19**	*D19Mit45*	(16.14)
	D3Mit12	(53.04)	**Chr 8**	*D8Mit171*	(11.43)					*D19Mit11*	(38.38)
	D3Mit160	(79.46)		*D8Mit65*	(24.98)	**Chr 13**	*D13Mit134*	(4.45)		*D19Mit34*	(51.90)
				D8Mit208	(49.80)		*D13Mit13*	(30.80)			
**Chr 4**	*D4Mit97*	(13.00)		*D8Mit167*	(83.92)		*D13Mit74*	(67.25)	**Chr X**	*DXMit89*	(4.73)
	D4Mit111	(28.52)					*D13Mit77*	(80.92)		*DXMit140*	(28.39)
	D4Mit17	(32.17)	**Chr 9**	*D9Mit296*	(14.38)					*DXMit147*	(41.80)
	D4Mit219	(48.22)		*D9Mit229*	(26.83)	**Chr 14**	*D14Mit206*	(11.53)		*DXMit13*	(62.40)
	D4Mit48	(76.38)		*D9Mit182*	(71.55)		*D14Mit207*	(16.74)			
				D9Mit18	(91.21)		*D14Mit59*	(32.60)			
**Chr 5**	*D5Mit193*	(2.36)					*D14Mit160*	(48.62)			
	D5Mit13	(21.92)					*D14Mit5*	(54.21)			
	D5Mit290	(39.36)					*D14Mit125*	(70.22)			
	D5Mit41	(58.98)					*D14Mit266*	(94.10)			
	D5Mit292	(98.00)									

The map positions of polymorphic markers were calculated from our QTL study; however, the map positions of most centromeric markers for each chromosome were obtained from the Mouse Genome Database (http://www.informatics.jax.org).

Supplementary Table S4. Polymorphic markers used in the congenic study

Chromosome	Marker	(cM)	Chromosome	Marker	(cM)	Chromosome	Marker	(cM)	Chromosome	Marker	(cM)
**Chr 1**	*D1Mit64*	(3.67)	**Chr 5**	*D5Mit148*	(17.33)	**Chr 11**	*D11Mit74*	(3.52)	**Chr 14**	*D14Mit206*	(11.53)
	D1Mit173	(20.14)		*D5Mit41*	(50.68)		*D11Mit76*	(9.41)		*D14Mit207*	(12.07)
	D1Mit19	(38.01)		*D5Mit262*	(70.66)		*D11Mit229*	(15.63)		*D14Mit209*	(15.06)
	D1Mit215	(39.91)					*D11Mit231*	(21.92)		*D14Mit186*	(16.80)
	D1Mit305	(44.95)	**Chr 6**	*D6Mit273*	(22.51)		*D11Mit236*	(27.39)		*D14Mit59*	(24.47)
	D1Mit218	(55.76)		*D6Mit178*	(42.99)		*D11Mit314*	(31.05)		*D14Mit5*	(31.49)
	D1Mit14	(67.71)		*D6Mit52*	(62.56)		*D11Mit242*	(39.47)		*D14Mit235*	(33.34)
	D1Mit269	(72.09)		*D6Mit14*	(77.64)		*D11Mit156*	(39.47)		*D14Mit125*	(44.17)
	D1Mit353	(76.84)					*D11Mit320*	(43.21)		*D14Mit266*	(64.86)
	D1Mit461	(89.95)	**Chr 7**	*D7Mit20*	syntenic		*D11Mit195*	(51.34)			
	D1Mit155	(98.20)		*D7Mit62*	(48.36)		*D11Mit286*	(54.95)	**Chr 15**	*D15Mit113*	(18.93)
				D7Mit238	(63.78)		*D11Mit70*	(58.90)		*D15Mit123*	(29.31)
**Chr 2**	*D2Mit2*	(4.24)					*D11Mit54*	(59.82)		*D15Mit42*	(55.72)
	D2Mit296	(21.81)	**Chr 8**	*D8Mit171*	(11.43)		*D11Mit145*	(61.07)			
	D2Mit37	(44.13)		*D8Mit208*	(42.45)		*D11Mit301*	(75.93)	**Chr 16**	*D16Mit88*	(9.23)
	D2Mit304	(62.49)		*D8Mit167*	(63.84)		*D11Mit168*	(78.74)		*D16Mit4*	(25.43)
	D2Mit51	syntenic								*D16Mit158*	(45.89)
			**Chr 9**	*D9Mit229*	(26.83)	**Chr 12**	*D12Mit270*	(13.69)			
**Chr 3**	*D3Mit117*	(1.96)		*D9Mit269*	(47.15)		*D12Mit255*	(37.82)	**Chr 17**	*D17Mit36*	(22.90)
	D3Mit98	(37.83)		*D9Mit311*	(68.74)		*D12Mit20*	(62.10)		*D17Mit206*	(45.20)
	D3Mit257	(66.69)									
			**Chr 10**	*D10Mit194*	(24.48)	**Chr 13**	*D13Mit13*	(30.06)	**Chr 18**	*D18Mit35*	(23.86)
**Chr 4**	*D4Mit111*	(28.7)		*D10Mit230*	(45.28)		*D13Mit74*	(56.92)		*D18Mit7*	(51.92)
	D4Mit219	(47.21)		*D10Mit164*	(67.32)		*D13Mit78*	(67.21)			
	D4Mit48	(73.41)							**Chr 19**	*D19Mit80*	(18.24)
										D19Mit34	(50.72)

The map positions of polymorphic markers were obtained from the Mouse Genome Database (http://www.informatics.jax.org). The positions of these markers are different from those obtained from the QTL study.

Supplementary Figure S1

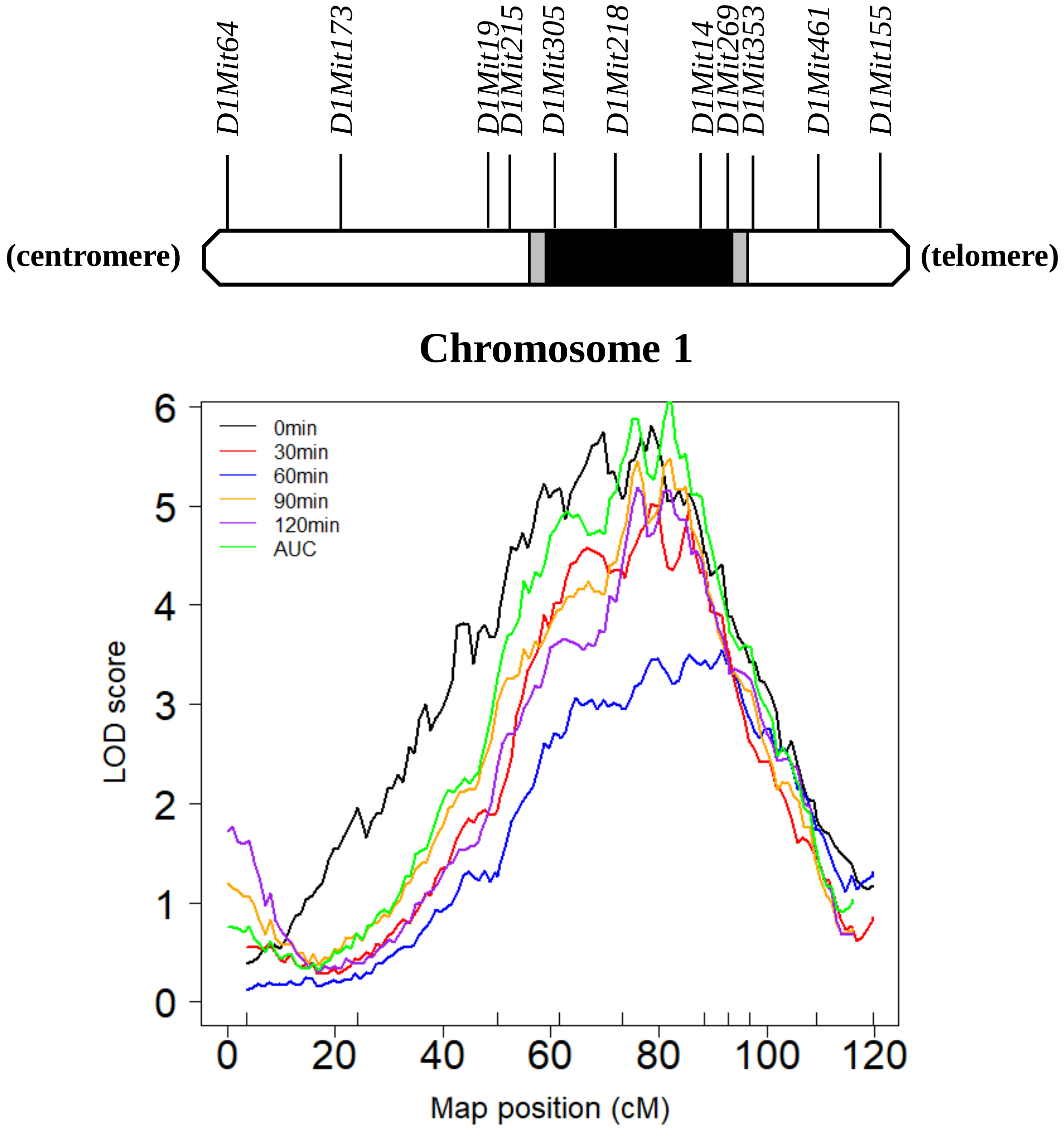

Supplementary Figure S2

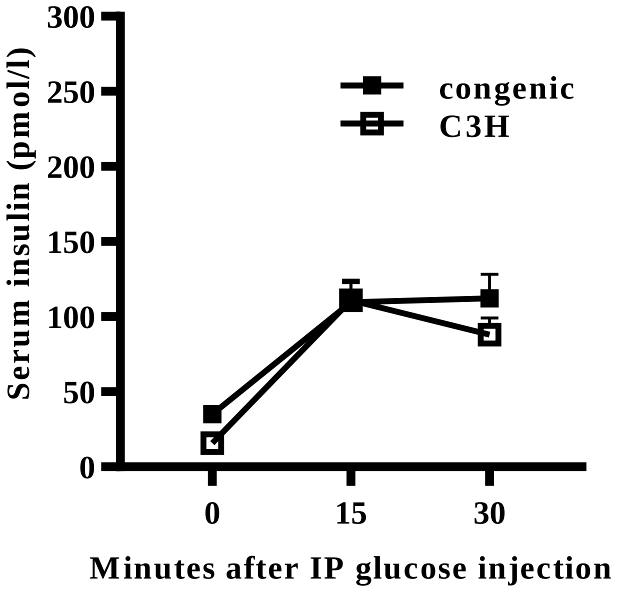

Supplementary Figure S3

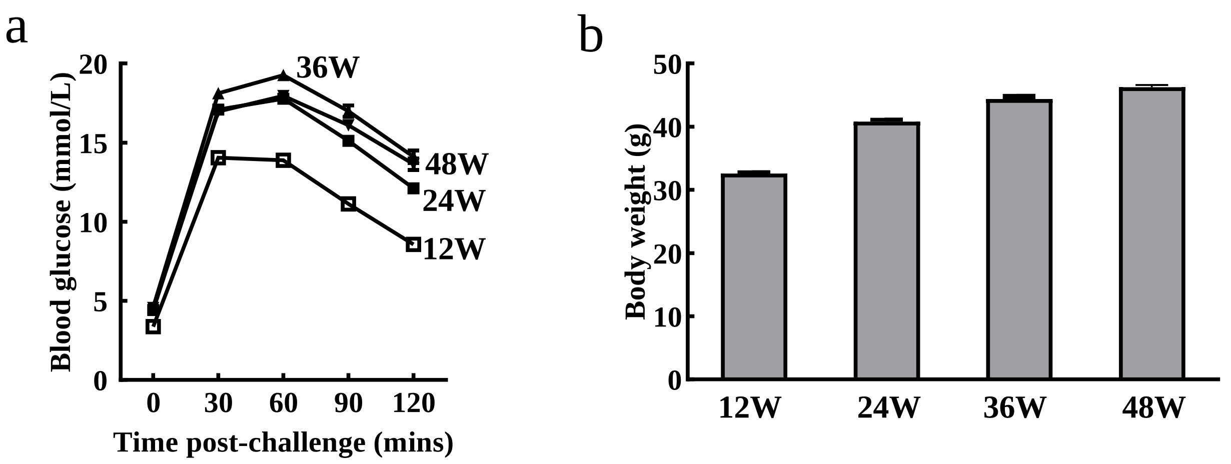

Supplementary Figure S4

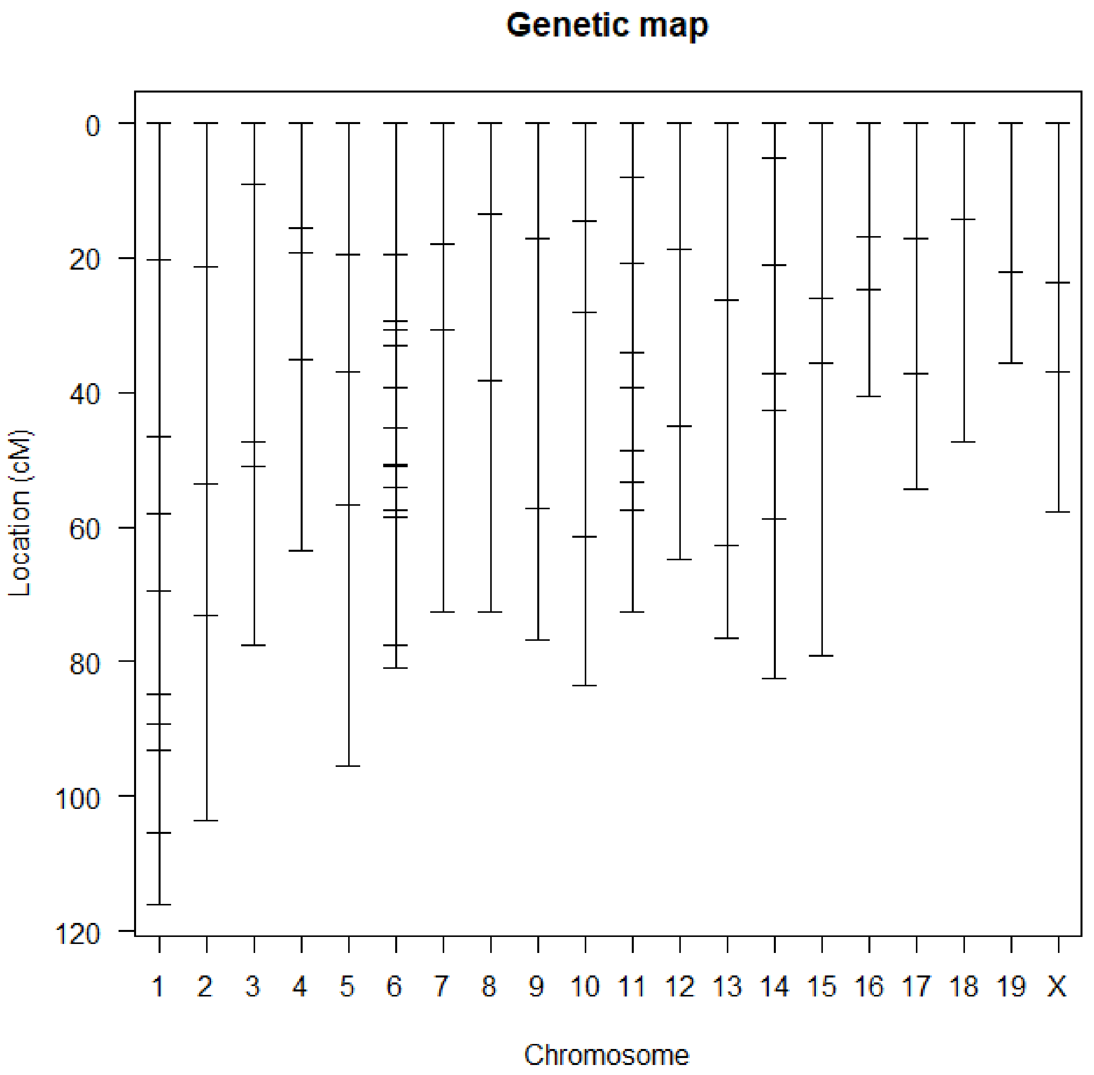

Supplementary Figure Legends

Supplementary Figure S1. Congenic interval derived from NSY chromosome 1 in C3H.NSY-*Nidd5nsy* mice (upper panel) relative to the LOD score curve for hyperglycemia (lower panel).

Supplementary Figure S2. Phenotypes of C3H.NSY-*Nidd5nsy* and C3H mice. Insulin secretion in response to glucose during ipGTT at 26 weeks of age.

Supplementary Figure S3. Phenotypes of F2 mice. Blood glucose during ipGTT (panel a) and body weight (panel b) at 12, 24 36 and 48 weeks of age.

Supplementary Figure S4. Chromosomal position of markers listed in supplementary Table S3.
